# Advances of nanotechnology for intracerebral hemorrhage therapy

**DOI:** 10.3389/fbioe.2023.1265153

**Published:** 2023-09-12

**Authors:** Jiayan Wang, Tianyou Wang, Mei Fang, Zexu Wang, Wei Xu, Bang Teng, Qijuan Yuan, Xin Hu

**Affiliations:** ^1^ Department of Neurosurgery, West China Hospital, Sichuan University, Chengdu, China; ^2^ West China School of Medicine, Sichuan University, Chengdu, China; ^3^ State Key Laboratory of Polymer Materials Engineering, College of Polymer Science and Engineering, Sichuan University, Chengdu, China; ^4^ School of Materials Science and Engineering, Xihua University, Chengdu, China

**Keywords:** intracerebral hemorrhage, therapeutic agents, nanotechnologies, safety, bioavailability, functionality

## Abstract

Intracerebral hemorrhage (ICH), the most devastating subtype of stoke, is of high mortality at 5 years and even those survivors usually would suffer permanent disabilities. Fortunately, various preclinical active drugs have been approached in ICH, meanwhile, the therapeutic effects of these pharmaceutical ingredients could be fully boosted with the assistance of nanotechnology. In this review, besides the pathology of ICH, some ICH therapeutically available active drugs and their employed nanotechnologies, material functions, and therapeutic principles were comprehensively discussed hoping to provide novel and efficient strategies for ICH therapy in the future.

## 1 Introduction

**FIGURE 1:**
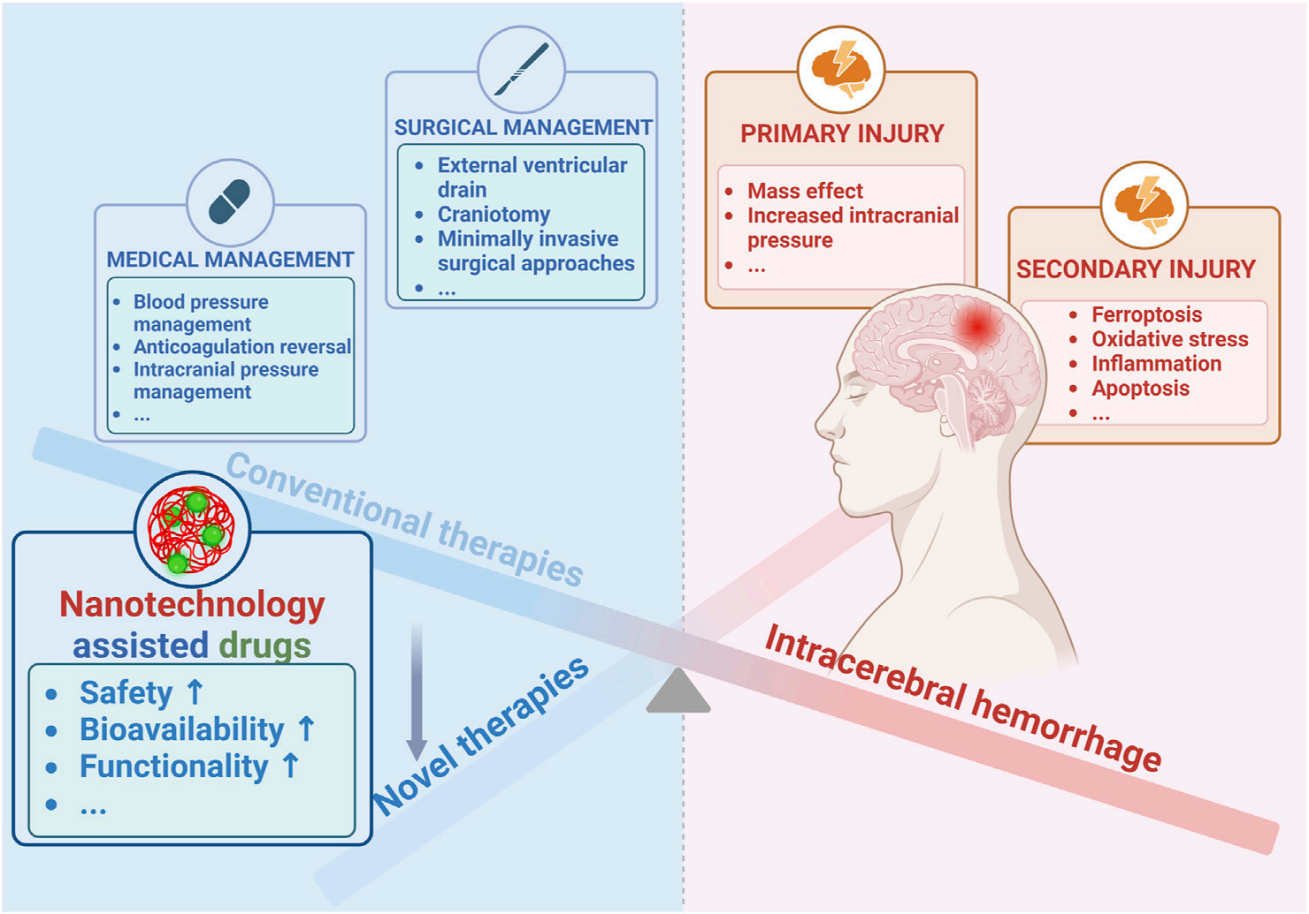
Advances of nanotechnology for ICH therapy. Created with Biorender.com.

Stroke is the second leading global cause of death and the third one of death and disability combined (Collaborators, 2021). Thanks to the progress in medicine, the situation of stroke patients has been improved overall (Elkind and Hankey, 2022), nevertheless, stroke is still a great threat to human particularly people in most areas of China where the prevalence of stroke consecutively increased from 2013 to 2019 (Tu et al., 2022). As the most serious subtype of stroke (Cordonnier et al., 2018a), intracerebral hemorrhage (ICH) affects about 2 million people worldwide annually (Krishnamurthi et al., 2013), and is critically life-threatening with a 1-year survival rate of less than 40% (Bejot et al., 2018). Extensive research efforts have led to a growing understanding of this disease, particularly its detrimental mechanisms including primary injury and secondary injury (Figure 1) (Wilkinson et al., 2018b; Campbell and Khatri, 2020; Bautista et al., 2021; Sheth, 2022). The primary injury after ICH is mainly referred to hematoma induced mass effect (Wilkinson et al., 2018a), and the subsequent secondary injury is more complex, including brain edema (Wan et al., 2023), ferroptosis (Wan et al., 2019), oxidative stress (Hu et al., 2016), inflammation (Xue and Yong, 2020), apoptosis (Chen et al., 2020), etc. Although ICH is rather detrimental, as shown in Figure 1, clinical approaches available in ICH are rather limited including medical managements such as blood pressure management, anticoagulation reversal, and intracranial pressure management (Cordonnier et al., 2018b; Kirshner and Schrag, 2021), as well as the surgical ones aiming to remove hematoma such as external ventricular drain, craniotomy, minimally invasive approaches, and so on (de Oliveira Manoel, 2020). However, with all of these treatments, the prognosis of ICH is still hard to be improved profoundly (Rabinstein, 2017). Accordingly, it is imperative to develop more therapeutic agents particularly those targeting the secondary injury, which is strongly associated to the outcomes of ICH (Bautista et al., 2021). In recent years, increasingly active pharmaceutical ingredients were applied against ICH in preclinical studies comprising deferoxamine (Xu et al., 2020b), curcumin (Yang C. et al., 2021), and resveratrol (Mo et al., 2021), etc., however, most of them could be hardly applied to ICH patients since their toxicity, poor bioavailability, and limited functions (Yu H. et al., 2023). Thus, it is practical to improve the intrinsic disadvantages of current drugs through rational design and novel drug loading techniques, particularly by exploiting the nanotechnology (Yu H. et al., 2023). Actually, nanomaterials such as polymer (Xu et al., 2020b), micelles (Zi et al., 2021), nanoemulsions (Marques et al., 2020), liposomes (Xu et al., 2020a), and exosomes (Hu et al., 2023), etc., have been widely designed and applied for ICH therapy, and their properties including biocompatibility, biodegradability, and engineerability, etc., have been roundly demonstrated (Figure 1) **(** Xu et al., 2022; Zhang et al., 2023). In this review, we mainly summarized the pathological mechanisms in ICH, potential therapeutic targets, and the role of nanotechnologies in drug design and delivery. The aim was to discuss the potential of nanotechnologies in enhancing therapeutic effects of drugs and to provide a better theoretical basis for supporting future therapies of ICH.

## 2 Injuries and mechanisms of ICH

**FIGURE 2:**
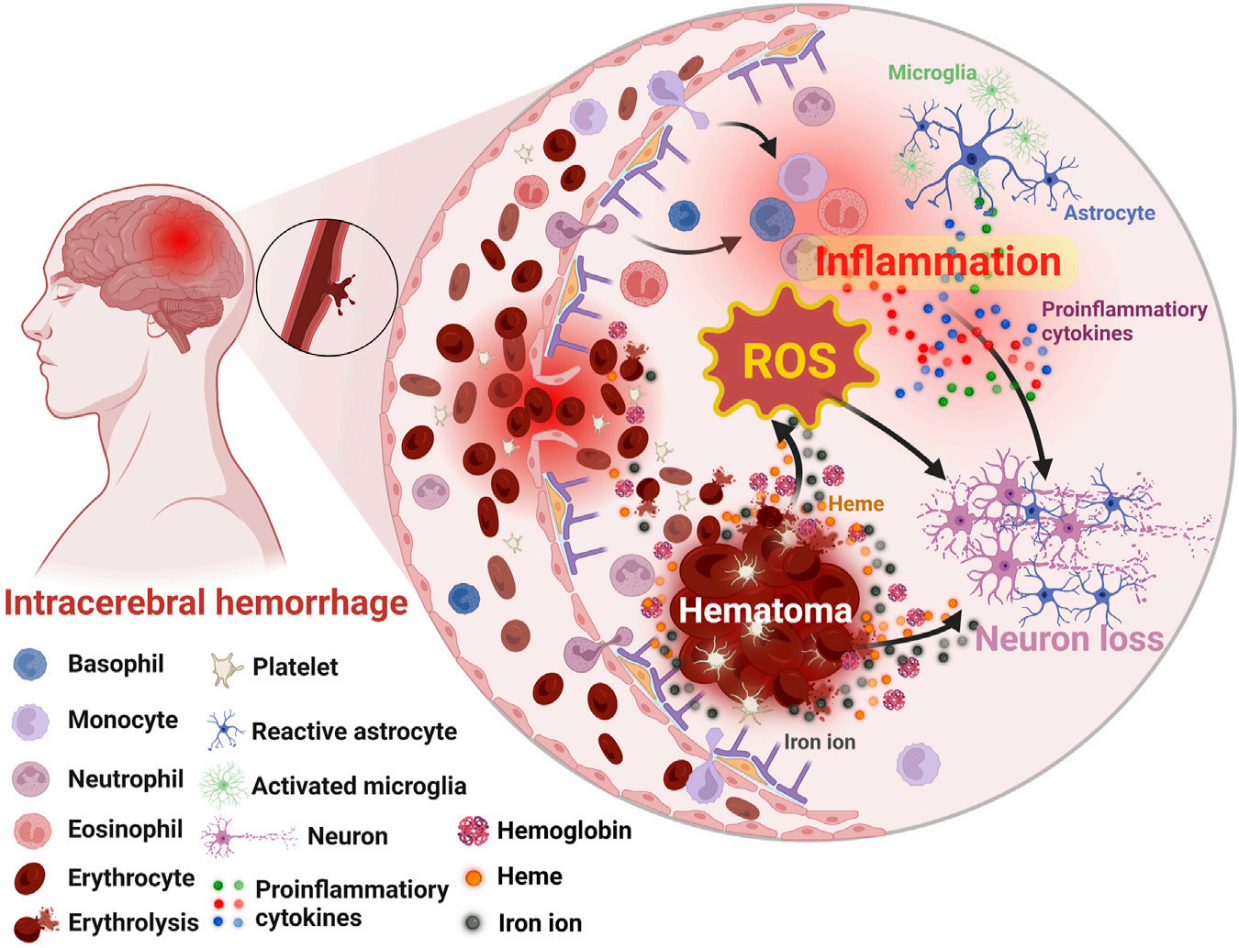
Schematic illustration of the secondary brain injury after ICH. Created with Biorender.com.

Generally, the damages caused by ICH could be divided into primary and secondary injuries (Puy et al., 2023). The former mainly consists of the hematoma induced mass effect, subsequent increased intracranial pressure, and so on (Wilkinson et al., 2018a; Kalisvaart et al., 2022; Wan et al., 2023). With the formation of hematoma (Wilkinson et al., 2018a), secondary brain injury such as ferroptosis (Wan et al., 2019), oxidative stress (Hu et al., 2016), inflammation (Xue and Yong, 2020), and apoptosis (Chen et al., 2020) gradually emerges (Figure 2). Specifically, ICH occurs when some brain vessels rupture, causing blood to flood into the brain parenchyma and form hematoma (Chang et al., 2018). With the accumulation of the blood, the hematoma might expand (Morotti et al., 2022) and the resultant mechanic compression as well as subsequent increased intracranial pressure would emerge gradually (Puy et al., 2023). Notably, the hematoma is a determining factor for the primary injury, and strongly associated with the subsequent secondary injury especially inflammation and oxidative stress (Xu et al., 2020a; Liu et al., 2022). Within several days after ICH, hemolysis will occur in the hematoma, leading to the release of hemolytic products including hemoglobin and heme, which wound exaggerate inflammation (Wang et al., 2021; Xia et al., 2022). Subsequently, the iron degenerated from heme acts as the catalyst in Haber-Weiss reaction (Zhu et al., 2021), generating overloaded reactive oxygen species (ROS) and leading to detrimental oxidative stress (Wan et al., 2019). Additionally, thrombin and serum proteins derived from hematoma will worsen edema and damages of blood-brain barrier (BBB) (Katsu et al., 2010; Urday et al., 2015). Thus, in clinical practice, the volume of hematoma is a widely used prognostic biomarker and its rapid resolution is vital and urgent for ICH patients (Broderick et al., 1993; de Oliveira Manoel, 2020). In addition to the well-studied oxidative stress and the inflammatory injury (Ohashi et al., 2023; Xu et al., 2023), the apoptosis and ferroptosis induced by ICH had drawn increasing attention and became potential therapeutic targets in recent years since their relationship with the poor neurological outcomes of ICH patients (Wan et al., 2019; Chen et al., 2020). Despite great advances in understanding the pathological process of ICH had been achieved, many mechanisms remain unclear, and effective therapies for ICH patients are still insufficient (Rabinstein, 2017; Kirshner and Schrag, 2021; Gu et al., 2022). Fortunately, by the efforts of researchers, more and more active pharmaceutical ingredients targeting ICH were developed in preclinical studies with the help of nanotechnology (Yu H. et al., 2023). Specifically, by combining nanomaterials, not only many therapeutic agents’ intrinsic disadvantages such as toxicity and poor bioavailability were improved, but also their therapeutic effects were enhanced profoundly (Xu et al., 2022; Zhang et al., 2023).

## 3 Nanomaterials in ICH

As previously mentioned, ICH is the most fatal stroke type and affects about 2 million people globally each year (Krishnamurthi et al., 2013). Unfortunately, there is a lack of effective therapies for ICH (Rabinstein, 2017). While minimally invasive surgery for ICH is widely accepted particularly in high-income countries, there are still controversial issues that need to be addressed such as the lack of sufficient evidence and the fact that not all patients are suitable candidates for the surgical approach (Wilkinson et al., 2018a). In addition to surgery, many researchers are dedicating their efforts to developing potential therapies against ICH including novel technologies utilization and emerging nanomaterials such as polymer (Xu et al., 2020b), micelles (Zi et al., 2021), nanoemulsions (Marques et al., 2020), liposomes (Xu et al., 2020a), and exosomes (Hu et al., 2023). Noteworthily, some approaches including polymerization, self-assembly and microfluidics play a significant role not only in the design and fabrication of novel drug, but also in improving the efficacy of therapeutic agents for ICH by addressing intrinsic disadvantages such as toxicity, poor bioavailability, and unsatisfactory therapeutic performance (Xu et al., 2022; Yu H. et al., 2023; Zhang et al., 2023). Specifically, in this review, the employed nanotechnological strategies mainly consist of the advanced drug loading techniques, post-synthetic modification (PSM), and self-assembly (Table 1). Specifically, the drug loading techniques, which provide vehicles for delivering drugs to targeted position, can change the drug pharmacokinetics, improve the drug efficacy, and reduce adverse effects (Wang W. et al., 2019; Huang et al., 2019; Mehryab et al., 2020; Yu H. et al., 2023). As one of the widely applied nanotechnologies, PSM refers to functionalization of the synthesized the nanomaterials such as the PEGylation (Xu et al., 2020b), which can improve the properties of the therapeutic agents for requirements (Mandal et al., 2021; Figueroa-Quintero et al., 2023). Moreover, self-assembly, another important method of nanotechnology, by employing which a thermodynamically stable nanostructure can be achieved via weak and polyvalent interactions (Li et al., 2018b). And this structure is of fast response to environmental stimuli since its low energy barrier (Stuart et al., 2010), which is of significance in nanomedicine (Srikanth and Kessler, 2012). Therefore, in the following section, some impressive recent works that focus on the role of nanomaterials in promoting the safety, bioavailability, and functions of therapeutic agents against ICH would be roundly introduced and discussed.

**TABLE 1:** Summary of current nanotechnological methods for ICH therapy.

Nanotechnologies	Material forms	Interactions	Active components	References
Drug loading	Polymer NPs	Hydrophilic and hydrophobic interaction	Curcumin	Yang et al. (2021a)
Drug loading	Polymer NPs	Hydrophilic and hydrophobic interaction	Resveratrol	Mo et al. (2021)
Drug loading	Nanomicelles	Hydrophilic and hydrophobic interaction	Rosuvastatin	Zi et al. (2021)
Drug loading	Nanoemulsions	Hydrophilic and hydrophobic interaction	Curcumin	Marques et al. (2020)
Drug loading	Liposomes	Hydrophobic interaction, hydrogen bond	IL-4	Xu et al. (2020a)
Drug loading	Exosomes	Hydrophobic interaction, hydrogen bond	miR-23b	Hu et al. (2023)
Drug loading	NPs	Absorption	Se	Yang et al. (2021b)
Drug loading	Nanoemulsions	Hydrophilic and hydrophobic interaction, hydrogen bond	Quercetin	Galho et al. (2016)
Drug loading	Liposomes	Hydrophobic interaction, hydrogen bond	CeNPs	Cha et al. (2018)
Drug loading	Polymer NPs	Hydrophobic interaction, hydrogen bond, electrostatic interaction	cmvNT-3-HRE complexes	Chung et al. (2013)
Drug loading	Nanomicelles	Hydrophobic interaction	Dauricine	Li et al. (2020)
Drug loading	DNA nanorobotics	Covalent bond, hydrogen bond	siCCR2	Fu et al. (2021)
Drug loading	Exosomes	Hydrophobic interaction, hydrogen bond	miR-133b	Shen et al. (2018)
Drug loading	Exosomes	Hydrophobic interaction, hydrogen bond	miR-146a-5p	Duan et al. (2020)
Drug loading	Core-shell hydrogel	Hydrophilic and hydrophobic interaction, hydrogen bond	EGF, bFGF	Gong et al. (2020)
PSM	Polymer NPs	Covalent bond	DFO	Xu et al. (2020b)
PSM	NPs	Hydrogen bond	Menp	Xu et al. (2023)
PSM	NPs	Hydrophobic interaction, coordination interaction	CeNPs	Zheng et al. (2021)
PSM	NPs	Hydrophobic interaction, coordination interaction	CeNPs	Kang et al. (2017)
PSM	Polymer NPs	Covalent bond	DFO-HCC-PEG	Dharmalingam et al. (2020)
Self-assembly	Polymer NPs	Covalent bond, Hydrophilic and hydrophobic interaction, hydrogen bond	DFO, poly (catechol)	Zhu et al. (2021)
Self-assembly	Aggregates	Covalent bond, Hydrophilic and hydrophobic interaction, hydrogen bond	ELP	Park et al. (2017)
Self-assembly	SAPNS	Hydrophilic and hydrophobic interaction, electrostatic interaction	RADA16-I	Sang et al. (2015)
Self-assembly	Polymer NPs	Covalent bond, Hydrophilic and hydrophobic interaction, hydrogen bond	TEMPO	Chonpathompikunlert et al. (2012)
Self-assembly	SAPNS	Hydrophilic and hydrophobic interaction, electrostatic interaction	RADA16mix	Zhang et al. (2016)
Self-assembly	Polymer NPs	Covalent bond, Hydrophilic and hydrophobic interaction, hydrogen bond	DFO, polyphenols	Zhu et al. (2023b)
Self-assembly	Polymer NPs	Covalent bond, Hydrophilic and hydrophobic interaction, hydrogen bond	DFO, natural polyphenols	Zhu et al. (2023a)

NPs, nanoparticles; IL-4, interleukin-4; miR, microRNA; Se, selenium; CeNPs, cerium oxide nanoparticles; cmv, cytomegalovirus; NT-3, neurotrophin-3; HRE, containing hormone response element; siCCR2, C-C chemokine receptor 2; EGF, epidermal growth factor; bFGF, basic fibroblast growth factor; PSM, post-synthetic modification; DFO, deferoxamine; Menp, polydopamine; HCC, hydrophilic carbon clusters; PEG, polyethylene glycol; ELP, elastin-like polypeptide; SPANS, self-assembling peptide nanofiber scaffold; RADA16-I, Ac-RADARADARADARADA-CONH2 R, arginine; A, alanine; D, aspartate; TEMPO, 2,2,6,6-tetramethylpiperidine-1-oxyl.

### 3.1 Safety

**FIGURE 3:**
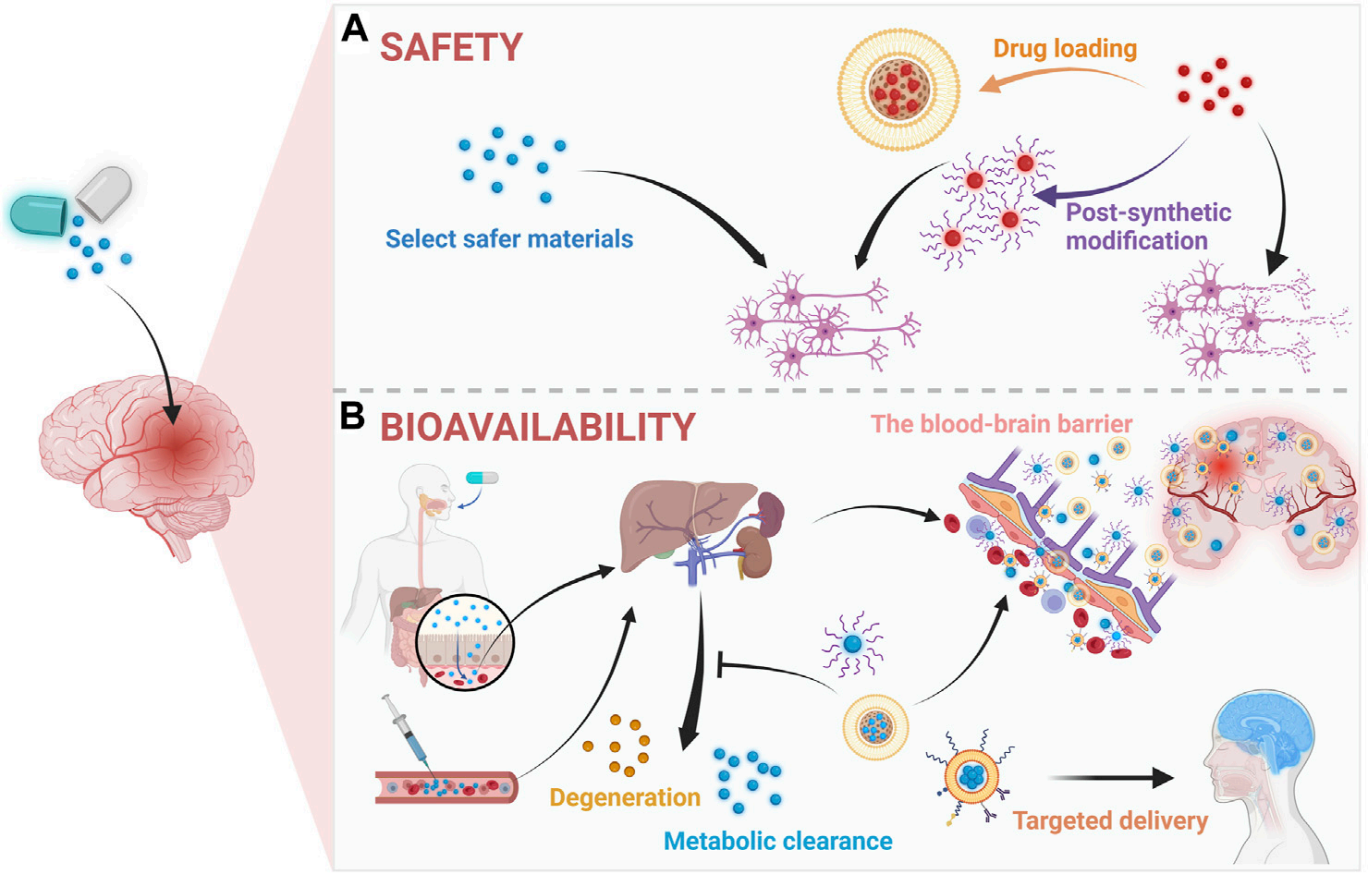
Application of nanotechnology in **(A)** safety and **(B)** bioavailability for therapeutic agents in ICH. Created with Biorender.com.

Like other drugs, the development of new therapeutic agents for ICH must prioritize safety to guarantee clinical application. Accordingly, safer pharmaceutical ingredients usually would be considered firstly. Besides, some therapeutic agents are of intrinsic toxicity such as selenium, whose therapeutic dose is close to toxic one and chronic selenium toxicity will damage major organs such as liver, spleen, and kidneys (Spallholz, 1994; Zhai et al., 2017; Yang Y. et al., 2021). And it is necessary to reduce toxicity with advance approaches such as nanotechnologies until meeting safety standards (Figure 3A). Specifically, through improving stability, controlled drug loading and release (Cha et al., 2018), non-specific distribution (Yu H. et al., 2023), and efficacy (Chung et al., 2013), etc., nanotechnology could render the drug administration in a low does and reduced frequency leading to less side-effects and better patient compliance (Xu et al., 2020b). Besides, the shape, size, and surface functionalization of drugs could be engineered by nanotechnology for more biocompatible and better medical application (Xu L. et al., 2020). For instance, inorganic materials with clear therapeutic effects like cerium oxide nanoparticles (CeNPs) have been studied and applied to ICH (Kang et al., 2017; Zheng et al., 2021) due to its superoxide dismutase mimetic and catalase mimetic properties for excess ROS scavenging (Bao et al., 2018; Zeng et al., 2018). To improve its biocompatibility, some researchers constructed new delivery systems such as the customized lipid-coated magnetic mesoporous silica nanoparticle doped with CeNPs (LMCs) (Cha et al., 2018). Briefly, CeNPs was loaded into biodegradable mesoporous silica nanoparticles (MSNs) and then capsulated by lipid bilayers. And the prepared LMCs with a surface charge (−1.35 mV) which is close to that of liposomes indicating the coating was successful and LMCs did demonstrate a great stability without agglomerations and retained the hydrodynamic size over a week. Moreover, with the high loading of CeNPs, LMCs demonstrated potent ROS scavenging capacity and could alleviate inflammation after ICH. In addition to inorganic nanomaterials, some organic materials such as polymer, nanoemulsion, and nanomicelles usually demonstrate low toxicity and biodegradability and are suit for clinical therapy, too. For instance, polyethyleneglycol (PEG) and polybutylcyanoacrylate (PBCA) are famous for low toxicity, non-immunogenicity, and stability, whereby they were usually employed to construct nanomaterials and applied in novel drug fabrication (Chung et al., 2013; Zheng et al., 2021). Specifically, through PEGylation, deferoxamine (DFO), the iron chelator (Holden and Nair, 2019), could be applied to remove excessive iron in ICH, whose cytotoxicity and poor hemocompatibility were improved effectively (Xu et al., 2020b). Specifically, in the cell viability assay, the cell viability of DFO group was 75% at a low concentration (0.05 mM) and decreased to 48.6% at a high concentration (0.5 mM), however, the PEGylated DFO groups demonstrated at least 85% cell viability at all concentration. Though the nanotechnology were extensively applied and elevated the biocompatibility of some drugs, the potential adverse effects or toxicity of the nanomaterials themselves should be concerned, too. There are some critical factors associated with the toxicity of nanomaterials including size, shape, surface charge, hydrophobicity, and the functional groups, however, surface modification techniques are effective in improving the biocompatibility of nanomaterials (Wei et al., 2023). As for central nervous systems, some metal-based nanomaterials such as copper, silver, and aluminum will damage BBB though it will facilitate drug delivery to brain after ICH (Zhang et al., 2023). Moreover, nanomaterials are easier in initiating responses of immune systems than small molecules and may damage organelles and DNA (Pallardy et al., 2017; Alexander et al., 2019). Thus, the safety of nanomaterials should be more considered and more investigation on their toxicity particularly after long term administration are necessary. And for the sake of safety, selecting more biocompatible materials, targeted delivery, and applying advanced technologies such as surface modification to improve the adverse effects of nanomaterials are promising.

### 3.2 Bioavailability

The ability of a kind of drugs or active pharmaceutical ingredients to exist at a certain concentration in target areas is commonly termed as bioavailability (Al-Kassas et al., 2017; Davis and Walker, 2018). Accordingly, the key to improve drug bioavailability is to maintain the enough drug concentration in the blood circulation or prolong their half-life. In recent years, as shown in Figure 3B, nanomaterials had contributed significantly to achieving this goal, for instance, by PEGylation the half-life of DFO was increased 20 folds in plasma (Xu et al., 2022; Yu H. et al., 2023; Zhang et al., 2023). And a common approach is to employ nanomaterials such as PEG and PBCA (Cha et al., 2018; Dharmalingam et al., 2020), by which the poor solubility and short half-time of drugs will be significantly improved such as the case of Resveratrol (Res) (Mo et al., 2021). Specifically, based on the methoxy poly (ethylene glycol)-poly (L-lactide-co-glycolide) (MPEG-PLGA), the Res-NPs was obtained through anti-solvent precipitation method. And by this, the poor water solubility, short half-life, and blood-brain barrier (BBB) penetration of Res were improved. Specifically, in the pharmacokinetic assessment, the Res-NPs demonstrated a higher half-life than Res in both of plasma (7.32 ± 0.65 h vs*.* 2.13 ± 0.27 h) and brain (11.36 ± 1.28 h vs*.* 7.95 ± 0.64 h). In addition to prolonging the half-life, targeted delivery approaches aiming to transport the drug to specific positions or cells are also highly promising (Yu W. et al., 2023). For example, rosuvastatin, a neuroprotective agent, is hard to directly use in ICH due to its toxicity and poor bioavailability (White, 2002). Nevertheless, encapsulating it in self-assembled nanomicelles basing poly (ethylene glycol)-block-poly (ε-caprolactone) (PEG-PCL) copolymers not only extended half-life of rosuvastatin, but also improved the targeted effects towards inflammation for lower dose and frequency (Zi et al., 2021). Specifically, the rosuvastatin nanomicelles demonstrated better efficacy in improving neurological deficit than rosuvastatin alone, which might be attributed to the better bioavailability of rosuvastatin nanomicelles since both of them are orally administrated with the same dose. Moreover, the advancement of nanotechnologies has enabled the integration of many building blocks into nanomaterials, leading to more efficient targeted drug delivery through selective binding between biomaterials such as antibodies and antigens. For instance, through modifying the nanomaterials with anti-CD47 antibodies to block CD47, the drug’s half-life could be extended by reducing clearance by phagocytes (Jaiswal et al., 2009), and more advantages in targeting tumor cells were demonstrated (Luo et al., 2023). Given the outstanding advantages of combining biomimetic functions derived from cell membrane with the flexibility of material chemistry, various types of cell membrane including leukocyte, erythrocyte, platelet, macrophage, and cancer cell, etc., disguised nanomaterials were constructed in recent years for different targets (Li et al., 2018). Noteworthily, cell membrane disguised nanomaterials will be recognized as autogenous cells *in vivo*, which effectively dampens the immune system elimination whereby the half-life in the circulation is prolonged. Specifically, by coated with macrophage membrane, not only the half-life of nanomaterials will be prolonged, but also the BBB penetration can be enhanced via membrane receptor-ligand interactions, which increases the bioavailability of therapeutic agents for ICH (Lopes et al., 2022). Additionally, by coating with platelet membrane, an effective targeted delivery system for ICH was established basing the biological function of platelet for targeting and repairing damaged vessels, whereby the platelet-membrane-coated polydopamine nanoparticles successfully improved ICH induced injuries (Xu et al., 2023). Thus, the combination of these new technologies and materials could facilitate the treatment of ICH by improving the bioavailability of therapeutic agents.

### 3.3 Potential targets in ICH and functionalities of nanomaterials

**FIGURE 4:**
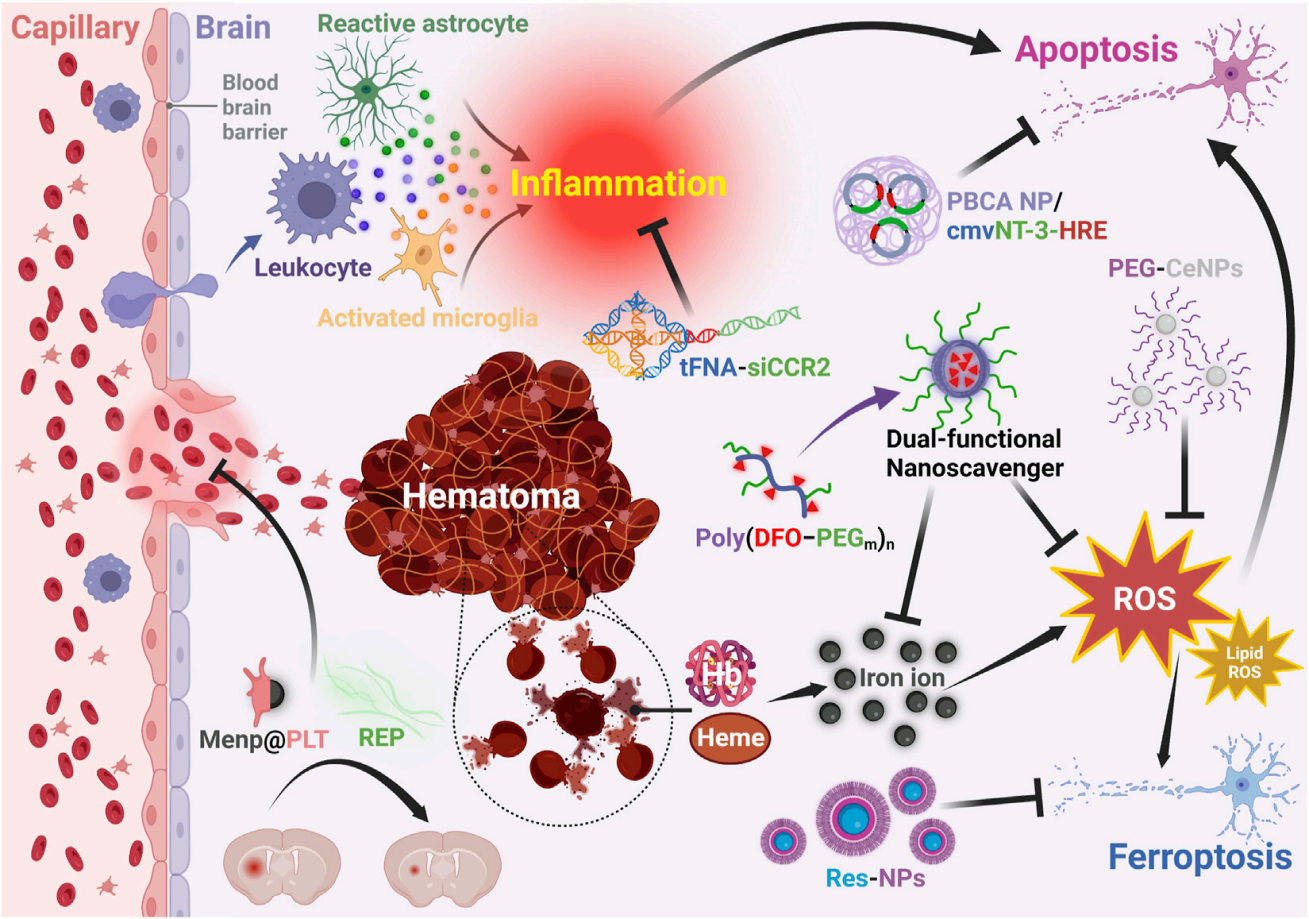
Functions of nanotechnology assisted therapeutic agents for ICH. PBCA: polybutylcyanoacrylate; NP: nanoparticle; cmv: cytomegalovirus; NT-3: neurotrophin-3; HRE: containing hormone response element; tFNA: tetrahedral framework nucleic acid; siCCR2: C-C chemokine receptor 2; PEG: polyethylene glycol; CeNPs: cerium oxide nanoparticles; DFO: deferoxamine; ROS: reactive oxygen species; Menp: polydopamine; PLT: platelet; REP: the repetitive integrin-binding ArgGlyAsp peptide -containing elastin-like polypeptide; Res: resveratrol. Created with Biorender.com.

Besides contributing to the improvement of the drug safety and bioavailability, nanotechnology also plays a crucial role in enhancing the therapeutic effects of drugs that target different pathological processes in ICH (Table 2). Specifically, as shown in Figure 4, therapeutic agents assisted by nanomaterials have demonstrated better performance in reducing hematoma (Park et al., 2017; Xu et al., 2023), ferroptosis (Mo et al., 2021), oxidative stress (Zheng et al., 2021), inflammation (Fu et al., 2021), and apoptosis (Chung et al., 2013) in ICH treatment.

**TABLE 2:** Summary of the application of nanotechnology assisted drugs in ICH.

Drug designs	Nanotechnologies	Functions	Therapeutic effects	References
PEGylated DFO	PSM	Increases half-life and stability; reduces cytotoxicity	Reduces excessive iron and neuronal degeneration; improves function recovery	Xu et al. (2020b)
Curcumin NPs	Drug loading	Improves half-life, water solubility, and efficacy	Reduces hematoma size and neurological deficits, and erastin-induced HT22 cell ferroptosis	Yang et al. (2021a)
Resveratrol NPs	Drug loading	Improve oral bioavailability and efficacy by increasing solubility in water, half-life, and BBB penetration	Inhibits ROS generation and ferroptosis induced by erastin in HT22 mouse hippocampal cells	Mo et al. (2021)
Rosuvastatin-loaded nanomicelles	Drug loading	Reduces toxicity and improves bioavailability by increasing water solubility and half-life	Reduces inflammatory cell infiltration, the expression of proinflammatory cytokines, edema, neuron degeneration; promotes the expression of anti-inflammatory cytokine, microglia/macrophages to M2 polarization, and functional recovery	Zi et al. (2021)
Curcumin-loaded nanoemulsion	Drug loading	Improves bioavailability by increasing water solubility, BBB penetration, and reducing metabolic clearance	Reduces hematoma size, weight loss, and oxidative stress; promotes brain antioxidant status and motor recovery	Marques et al. (2020)
IL-4 protein-loaded liposomes	Drug loading	Improves IL-4 brain penetration and reduces systemic bystander effects with the help of nasal delivery	Promotes hematoma resolution and long-term functional recovery by IL-4/STAT6/ST2 pathway	Xu et al. (2020a)
BMSCs-exosomal miR-23b	Drug loading	Facilitates genetic molecular transferring	Reduces neurological deficits, apoptosis, PTEN/Nrf2 pathway mediated oxidative stress, and NLRP3 inflammasome-mediated pyroptosis	Hu et al. (2023)
Dual-functional macromolecular nanoscavengers	Self-assembly	Reduces toxicity and side-effect and realizes multifunction	Inhibits cell death by reducing excessive iron and ROS	Zhu et al. (2021)
Menp@PLT	PSM	Realizes the target delivery	Improves neuroinflammation environment by reducing ROS; reduces cell death, BBB permeability, and edema; decreases hematoma size by repairing damaged vessels	Xu et al. (2023)
Se@SiO_2_	Drug loading	Reduces toxicity by controlled releasing	Improves neurological function, glutathione peroxidase activity and malonaldehyde levels; reduces apoptosis, edema, and BBB damages	Yang et al. (2021b)
Quercetin-loaded nanoemulsions	Drug loading	Improves bioavailability by reducing lipophilicity	Improves the activity and content of GST, promotes motor functions, and reduces hematoma size and weight loss	Galho et al. (2016)
LMCs	Drug loading	Improves efficacy and colloidal stability by controlled loading and release, and lipid bilayers coating	Reduces ROS, inflammatory macrophage infiltration, and edema; improves neurologic outcomes	Cha et al. (2018)
PBCA NPs/cmvNT-3-HRE complexes	Drug loading	Reduces degradation and improves BBB penetration	Increases expression of NT-3; reduces apoptosis-inducing factor, cleaved caspase-3, DNA fragmentation, and cell death	Chung et al. (2013)
PEG-CeNPs	PSM	Improves biocompatibility and reduces interparticle agglomeration	Inhibits M1 microglia and A1 astrocyte activation by reducing ROS-induced NF-κB p65 translocation, and promotes OPC differentiation and remyelination	Zheng et al. (2021)
Phospholipid-PEG-capped CeNPs	PSM	Increases biocompatibility, half-life, and efficacy; reduces agglomeration and nonspecific dispersion	Reduces ROS, RNS, hemin-induced COX-2 expression, microglia/macrophage recruitment, cell death, and edema	Kang et al. (2017)
DFO-HCC-PEG	PSM	Increases half-life and cellular uptake; reduce toxicity; realizes multifunction	Reduces hemin- and iron-mediated neurotoxicity, senescence, and ferroptosis	Dharmalingam et al. (2020)
REP	Self-assembly	Improves cell adhesiveness and biological function	Reduces hematoma size by blocking damaged vessels; inhibits the leakage of IgG, microglia activation, and the expression of vWF	Park et al. (2017)
RADA16-I	Self-assembly	Improves biocompatibility	Reduces brain cavity formation by replacing hematoma, and improves sensorimotor functional recovery	Sang et al. (2015)
RNPs	Self-assembly	Reduces nonspecific dispersion and preferential renal clearance	Reduces ROS, edema, and neurologic deficit	Chonpathompikunlert et al. (2012)
Dauricine-loaded *p*-PCa4C12 micelles	Drug loading	Reduces metabolic clearance and realizes metal ion-responsive targeted delivery	Improves BBB integrity, edema, and neurological deficits; reduces neuroglia activation, neutrophils infiltration, pro-inflammatory factors, MMP-9, and ZO-1	Li et al. (2020)
FION-labeled SNMs	Drug loading	Realizes targeted delivery	Reduces recruitment of macrophages and neutrophils, and pro-inflammatory cytokines; improves neurological function and facilitates the stem cells transportation	Kang et al. (2020)
RADA16mix	Self-assembly	Improves biocompatibility by reducing acidity	Inhibits apoptosis, glial reaction, and inflammation; promotes nerve fibers growth and functional recovery	Zhang et al. (2016)
tFNA-siCCR2	Drug loading	Improves biocompatibility and stability	Inhibits CCR2 gene expression, reduces hematoma by promoting microglia M2 polarization, suppresses neuroinflammation by reducing inflammatory mediators and increasing anti-inflammatory factors, and improves neurological motion functions	Fu et al. (2021)
miR-133b exosomes	Drug loading	Facilitates genetic molecular transferring	Inhibits neuronal apoptosis and neurodegeneration by reducing RhoA expression and activating ERK1/2/CREB	Shen et al. (2018)
BMSCs-miR-146a-5p-exosomes	Drug loading	Facilitates genetic molecular transferring	Reduces apoptosis, inhibits inflammation by reducing pro-inflammatory mediators and microglia/macrophages M1 polarization, and improves neurological function	Duan et al. (2020)
DFO NPs	Self-assembly	Inhibits metabolic clearance and realizes multifunction	Reduces excessive iron, ROS, and pro-inflammatory cytokines	Zhu et al. (2023b)
Polyphenol-DFO NPs	Self-assembly	Increases half-life and stability, and reduces nonspecific toxicity	Reduces excessive iron and ROS	Zhu et al. (2023a)
EGF/bFGF-loaded PLGA nanoparticle-encapsulated core-shell hydrogel	Drug loading	Facilitates stem cell transplantation and realizes multifunction	Reduces excessive iron and edema, facilitates stem cell transplantation and neurological recovery	Gong et al. (2020)

PEG, polyethylene glycol; DFO, deferoxamine; PSM, post-synthetic modification; NPs, nanoparticles; ROS, reactive oxygen species; BBB, the blood-brain barrier; IL-4, interleukin-4; STAT6, signal transducer and activator of transcription 6; ST2, IL-1 receptor-like 1; BMSC, bone marrow mesenchymal stem cell; miR, microRNAs; PTEN, chromosome 10; Nrf2, nuclear factor erythroid-2-related factor 2; NLRP3, nucleotide-binding oligomerization domain-like receptor family pyrin domain containing 3; Menp, polydopamine; PLT, platelet; Se, selenium; GST, glutathione S-transferase; LMCs, lipid-coated magnetic mesoporous silica nanoparticles doped with ceria nanoparticles; PBCA, polybutylcyanoacrylate; cmv, cytomegalovirus; NT-3, neurotrophin-3; HRE, containing hormone response element; CeNPs, cerium oxide nanoparticles; NF-κB, nuclear factor-κB; OPC, oligodendrocyte progenitor cell; RNS, reactive nitrogen species; COX-2, cyclooxygenase-2; HCC, hydrophilic carbon clusters; REP, the repetitive integrin-binding ArgGlyAsp peptide -containing elastin-like polypeptide; IgG, immunoglobulin G; vWF, von Willebrand factor; RADA16-I, Ac-RADARADARADARADA-CONH2 R, arginine; A, alanine; D, aspartate; RNPs, nitroxide radical-containing nanoparticles; MMP-9, matrix-metalloprotease-9; ZO-1, zonula occludens-1; FIONs, magnetosome-like ferromagnetic iron oxide nanocubes; SNMs, spherical neural masses; tFNA, tetrahedral framework nucleic acid; siCCR2, C-C chemokine receptor 2; ERK1/2, extracellular signal regulating kinase; CREB, cAMP, response element-binding protein; EGF, epidermal growth factor; bFGF, basic fibroblast growth factor.

#### 3.3.1 Hematoma

As aforementioned, the hematoma size dictates the outcomes of ICH patients to a great extent (Hanley et al., 2019). Although large hematomas could be eliminated by various surgeries, many patients are not suitable for surgical management due to various reasons (Wilkinson et al., 2018a). Specifically, surgery is deemed of life saving in supratentorial ICH, however, the benefits of surgery are not clear for stable ICH patients or those in coma (Hemphill et al., 2015; Cordonnier et al., 2018b). Further, limited by the deep location of hematoma in the brain for some ICH patients and potential complications of surgery, removing hematoma by surgery failed to demonstrate significantly improved outcomes after ICH (Mendelow et al., 2005). Therefore, many nanomaterials based drugs targeting hematoma resolution were developed such as IL-4 protein loaded liposome NPs, which could facilitate hematoma resolution and function recovery after ICH through IL-4/STAT6/ST2 signaling (Xu et al., 2020a). Besides, platelet-membrane-modified polydopamine NPs (Menp@PLT) also reduced hematoma size after ICH by stopping bleeding through repairing the damaged vessels after ICH (Xu et al., 2023). Noteworthily, the Menp@PLT reduced more hematoma volume than Menp alone at 3 days after ICH. For instance, based on a thermally responsive biopolymer, modified elastin-like polypeptide (ELP), ArgGlyAsp (RGD) peptide containing ELP (REP) was fabricated (Park et al., 2017). And after ICH, REP reduced more hematoma volume significantly than RGD or ELP alone within 48 h after ICH through covering the broken vessels and preventing further bleeding. Thanks to it, not only less blood components were leaked, but also the activation of microglia and the expression of von Willebrand Factor (vWF) were inhibited, which led to reduced injuries in ICH. Moreover, by combining surgical hematoma aspiration with intrastriatally administrated RADA16-I (Sang et al., 2015), the hematoma volume was significantly reduced, and the residual cavity could be replaced that facilitated the neuron protection and sensorimotor functional recovery. In this case, the RADA16-I, a kind of self-assembled peptide nanofiber scaffolds (SAPNS), could form stable *β*-sheet structures which could further transform into hydrogels due to its regular repeats of ionic hydrophilic and hydrophobic amino acids (Schneider et al., 2008). Owing to this, the SAPNS was proven to provide supportive effects to various mammalian cells such as rat neuronal cells (Holmes et al., 2000; Kisiday et al., 2002), and it could facilitate the recovery of brain damages induced by hematoma in ICH. Noteworthily, there are other hematoma reducing therapeutic agents such as vitamin D (Liu et al., 2022) and bexarotene (Chang et al., 2020), whose effects are also hopefully being enhanced by exploiting nanocarriers, self-assembled nanomaterials, and so on.

#### 3.3.2 Ferroptosis

With the time passed, the erythrocytes in the hematoma will break down leading to an enormous release of hemoglobin (Hb), heme, and so on (Wang et al., 2021). Further degradation of heme causes the release of overwhelming free iron, resulting in iron overload and dysregulation of iron metabolism (Garton et al., 2016). This leads to the exaggeration of secondary brain injuries particularly ferroptosis, a newly identified programmed cell death that exacerbates neurological outcomes in ICH patients (Xie et al., 2016). To alleviate this condition, researchers extensively studied and utilized some pharmaceutical ingredients such as curcumin (Yang C. et al., 2021) and resveratrol (Res) (Mo et al., 2021) based nanomaterials to attenuate ferroptosis development after ICH through suppressing ROS generation and providing neuroprotective effects. For example, to gain better efficacy of Res, nanotechnology was employed to alleviate its poor water solubility, short half-life, and poor BBB penetration. Specifically, by embedding the Res in the MPEG-PLGA based polymer nanomaterials via an antisolvent precipitation method, more bioavailable Res-based NPs were obtained and demonstrated elevated efficacy in suppressing erastin induced ferroptosis in HT22 mouse hippocampal cells, in which the Res-NPs exerted more neuroprotection than Res alone demonstrated in both of cell viability assay and LDH release assay (Mo et al., 2021). Additionally, given the iron play a pivotal role in ferroptosis after ICH, directly removing the excessive iron after ICH is also a promising strategy (Wan et al., 2019). Thus, it is reasonable to further develop iron chelator such as DFO (Xu et al., 2020b) and minocycline (Cao et al., 2018) based nanomaterials against ICH induced ferroptosis.

#### 3.3.3 Oxidative stress

Oxidative stress, characterized by ROS, is a critical threat and a significant therapeutic target in ICH. Excessive ROS derives from various sources including mitochondria dysfunction, Hb-Heme-Iron, and inflammatory factors (Hu et al., 2016). To relieve oxidative stress and maintain the redox balance, nanomaterials including BMSC-exosomal miR-23b (Hu et al., 2023), Se@SiO_2_ nanocomposite (Yang Y. et al., 2021), LMCs (Cha et al., 2018), CeNPs (Zheng et al., 2021), quercetin-loaded nanoemulsion (Galho et al., 2016), and curcumin-loaded nanoemulsion (Marques et al., 2020) based therapies were developed in recent time. Among these, CeNPs were increasingly applied as potent scavengers of ROS and reactive nitrogen species (RNS), and demonstrated outstanding neuroprotective effects (Kim et al., 2012; Mracsko and Veltkamp, 2014). By passing through the damaged BBB in the hemorrhagic hemisphere, CeNPs effectively reduced oxidative stress, hemin-induced cytotoxicity, and inflammatory responses particularly the microglia/macrophage recruitment and inflammatory mediates release that leads to less brain edema (Kang et al., 2017). Moreover, drug loading or PSM were employed to satisfy more practical demands. For example, CeNPs modified with PEG (PEG-CeNPs) possessed better biocompatibility, less interparticle agglomeration, and success in improving white matter injury after ICH (Zheng et al., 2021). Additionally, loading CeNPs into the MSNs and coating them with a lipid bilayer not only realized the regulation of the loading and release of CeNPs, but also significantly strengthened their biocompatibility and functions (Cha et al., 2018). Apart from CeNPs, the 2,2,6,6-tetramethylpiperidine-1-oxyl (TEMPO) is famous for its potent ROS scavenging capacity as well (Samuni et al., 1988; Hahn et al., 1995), whereas its problems including nonspecific dispersion in normal tissues, fast degradability, and rapid renal clearance remain to be solved before its application in ICH. To this end, based on PEG-b-poly [(TEMPO) amino-methylstyrene] (PMNT) and PEG-b-poly [(TEMPO) oxy-methylstyrene] (PMOT), two kinds of redox polymer self-assembled nitroxide radical-containing nanoparticles (RNPs) were fabricated and demonstrated better bioavailability and great performances in reducing oxidative stress, brain edema, and neurologic deficits, which might be attributed to their longer half-life in plasma and brain than TEMPO (Chonpathompikunlert et al., 2012). Additionally, from the perspective of reducing oxidative stress without breaking the redox balance in normal tissues, it is necessary to develop target nanomaterials such as ROS responsive ones to improve the nonspecific dispersion. Specifically, the therapeutic agents would be released when the nanomaterials meet excessive intracellular ROS whereby a targeted delivery system was established (Yuan et al., 2021; Guo et al., 2023). Additionally, as mentioned before, through the function of platelet for targeting and repairing damaged vessels, polydopamine nanoparticles platelet-membrane-coated polydopamine nanoparticles were precisely delivered to the hemorrhage site after ICH and successfully exerted neuroprotection against oxidative stress (Xu et al., 2023). Given microglia and macrophage play great roles in ICH, targeting them to improve outcomes of ICH through a targeted therapy such as a phosphatidylserine liposome-based nanoparticle system is also worth to be considered (Han et al., 2023).

#### 3.3.4 Inflammation

Inflammatory responses, including the release of various cytokines and the recruitment of inflammatory cells, are common and important in injuries. However, overreacted inflammation will exacerbate the primary diseases (Hajishengallis and Chavakis, 2019). Accordingly, it is of great significance to take measures to palliate inflammatory responses properly in ICH. Several nanomaterials including rosuvastatin-loaded nanomicelles (Zi et al., 2021), dauricine-loaded *p*-PCa4C12 micelles (Li et al., 2020), iron oxide NPs-loaded human embryonic stem cell-derived spherical neural masses (Kang et al., 2020), and RADA16mix (Zhang et al., 2016) have been developed to target the inflammation. For instance, C-C chemokine receptor 2 (CCR2) is well-known for its roles in inflammatory diseases (Raghu et al., 2017; Tan et al., 2019; Pedragosa et al., 2020), and down-regulating its expression is deemed as a promising target in inflammation after ICH. To achieve this, tFNA-siCCR2 was established by loading siCCR2 into the tetrahedral framework nucleic acid (tFNA), a nanorobotic of editability, biocompatibility and low toxicity. And after intraventricularly administrated, the neuroinflammation in a mouse model of ICH was attenuated through regulating the polarization of microglia from M1 to M2, inhibiting the release of inflammatory mediators, and elevating the expression of anti-inflammatory factors. It led to accelerated hematoma absorption and partial preservation of motor nerve function (Fu et al., 2021). Since the complex effects of inflammation and its duration in ICH (Ohashi et al., 2023), it is important to properly realize the inflammation resolution instead of inhibiting inflammatory responses roughly (Kourtzelis et al., 2019). To this end, further explore drug administration in a controlled releasing method basing nanotechnology might be more appropriate in treating ICH (Hsu and Almutairi, 2021).

#### 3.3.5 Apoptosis

Apoptosis, in addition to its significant roles in physiological conditions, is deeply involved in diseases including ICH (Chen et al., 2020). After the onset of the ICH, the deterioration of the secondary injury results in increased cell death particularly the neuronal apoptosis, which is related to poor prognosis such as neurological deficits and disabilities (Puy et al., 2023). Thus, nanomaterials were being developed as potential approaches to alleviate neuronal apoptosis in ICH, including polymer (Chung et al., 2013) and exosomes (Shen et al., 2018; Duan et al., 2020). For instance, previous studies have reported that neurotrophin-3 (NT-3) had neuroprotective effects particularly in apoptosis models (Bouzas-Rodriguez et al., 2010), neurogenesis (Hao et al., 2017), and differentiation of stem cells (Yang et al., 2010). And some researchers employed cytomegalovirus (cmv) promoter controlled plasmid NT-3 containing hormone response element (HRE) to boost gene regulation and the increased NT-3 reduced ICH induced neuronal apoptosis (Chung et al., 2013). Specifically, polybutylcyanoacrylate nanoparticles (PBCA NPs) were taken as the drug delivery system due to great stability, bioavailability, low toxicity, and non-immunogenicity, which facilitated BBB penetration and prevented rapid degradation of the therapeutic agent. Consequently, the enrichment of PBCA NP/cmvNT-3-HRE complexes in the brain resulted in increased expression of NT-3, which led to significant inhibition of apoptosis-inducing factor, cleaved caspase-3, and DNA fragmentation. By these, the neuronal loss after ICH was effectively reduced by PBCA NP/cmvNT-3-HRE complexes whose neuroprotection was better than cmvNT-3-HRE reflected by both of TUNEL staining and Nissl staining. Pathologically, both of inflammation and oxidative stress will aggravate the neuronal apoptosis (Tian et al., 2023), thus, further developing multifunctional therapeutic agents which could inhibit inflammation, oxidative stress, and apoptosis simultaneously would be reasonable and effective.

#### 3.3.6 Multifunction

Although the aforementioned approaches effectively alleviated the damages in ICH, most of them target a single aspect. However, after ICH, various injuries with complex mechanisms are interconnected and mutually influenced (Bautista et al., 2021). Accordingly, there is a practical and necessary demand to develop some multi-targeted therapies that can simultaneously address different injuries. One example is the use of core-shell hydrogel based multifunctional nanomaterials, which could reduce excessive iron as well as effectively support stem cell-based therapy in ICH (Gong et al., 2020). Noteworthily, deferoxamine (DFO), a naturally siderophore applied in improving iron overload related diseases (Camaschella et al., 2020), has been increasingly integrated into multifunctional nanomaterials construction. For instance, DFO-hydrophilic carbon clusters (HCC)-PEG nanomaterials were developed to target both cellular senescence and ferroptosis after ICH (Dharmalingam et al., 2020). Additionally, by employing the 3,4-dihydroxyhydrocinnamic acid-DFO conjugate (Cat-DFO) as the building block, the polymeric DFO [poly (DFO)_n_] was then fabricated through horseradish peroxidase (HRP)- and hydrogen peroxide (H_2_O_2)_-catalyzed polymerization (Zhu et al., 2021). In which, by modifying the water-insoluble polymeric DFO [poly (DFO)_n_] with by PEG_2k_-NH_2_, it was successfully transformed into amphiphilic [poly (DFO-PEG_m_)_n_] with self-assembly property. This dual-functional nanoscavenger effectively decreased much more iron and ROS than DFO alone, resulting in reduced cell death. To further develop the DFO based multi-target nanomaterials, polyphenols with ROS-scavenging capacity were incorporated into carrier-free nanoparticles with high DFO-loading (∼80%) (Zhu et al., 2023b). The participation of polyphenols not only prolonged the drug’s half-life by restricting DFO’s metabolic clearance, but also realized better protection for brain cells after ICH due to the capacity of scavenging both excessive iron and ROS. Similarly, boronic acid modified DFO (PBA-DFO) was further modified with a series of natural polyphenols, resulting in natural polyphenols-boosted DFO NPs through supramolecular assembly (Zhu et al., 2023a). Importantly, this work presented a general strategy in which uniform and stable nanoparticles could be generated by the combination of natural polyphenols moiety and DFO. The NPs in this strategy not only removed excessive iron but also eliminated excessive ROS and attenuated inflammatory responses after ICH. This shed light on the promising prospect of multi-target nanomaterials in ICH. Overall, these complicated works with multi-target drug designs are insufficient in ICH. Apart from iron overload and oxidative stress, other pathological process in ICH comprising hematoma, edema, inflammation, ferroptosis, apoptosis, function recovery, etc., as targets are also worth to be integrated in multifunctional drugs design basing on nanotechnology. Notably, the functional recovery after ICH also is intractable. Thus, combining therapeutic nanomaterials with functional recovery therapies such as stem cell-based therapy is of potential, too (Gong et al., 2020). Further, given the imaging based diagnosis are quite important in ICH (Cordonnier et al., 2018b), it is necessary to develop theragnostic agents through incorporating with advanced nanomaterials based imaging technology (Ran and Xue, 2018; Szwargulski et al., 2020).

## 4 Conclusion and perspectives

**FIGURE 5:**
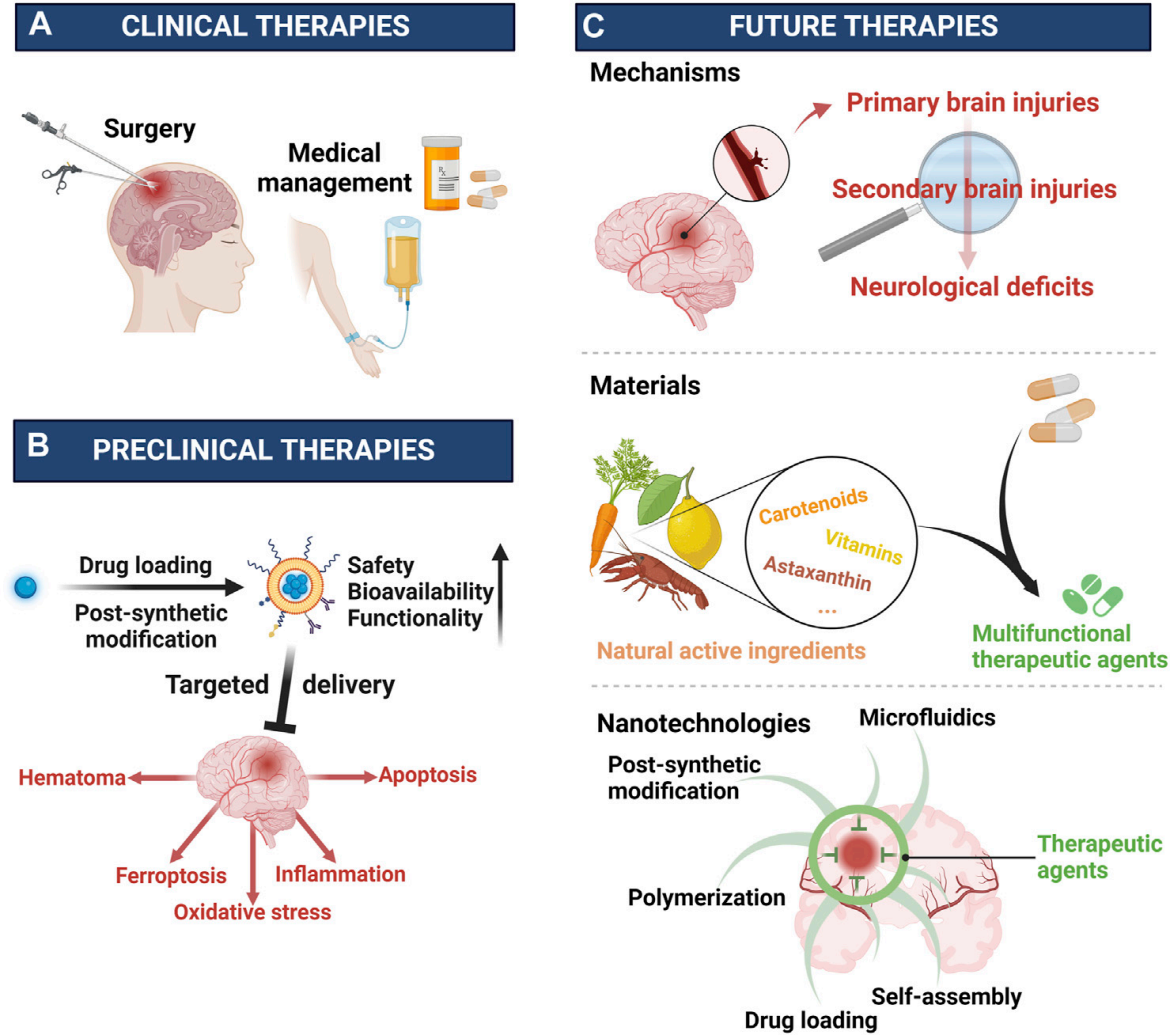
Clinical, preclinical, and future therapies for ICH **(A)** Clinical therapies are surgery and medical management. **(B)** Preclinical therapies are improved by nanotechnologies including drug loading, post-synthetic modification, etc. **(C)** Future therapies need to explore more pathological mechanisms underlying ICH, fabricate more multifunctional materials, and develop more advanced technology such as nanotechnology. Created with Biorender.com.

In addition to conventional therapies against ICH (Figure 5A), based on the comprehensive illustration of outstanding works targeting ICH, this review primarily focuses on the role of nanotechnology in novel drugs development particularly in the safety, bioavailability, and functionality (Figure 5B). Safety, as a cornerstone of the drug development, can be ensured through two approaches: selecting safe materials and reducing the drug toxicity with advanced technologies typified by nanotechnologies such as drug loading techniques and PSM. For the bioavailability, the drug’s half-life in blood determines the dosage and administration frequency. However, many potential drug candidates have poor bioavailability due to insolubility in water, fast metabolic clearance in the circulation, first pass effect and difficulty in passing the physiological barriers (Xu et al., 2022; Yu W. et al., 2023; Zhang et al., 2023). To address these challenges, various nanomaterials fabricated through polymerization, self-assembly and microfluidics were employed to modify or carry drugs pursuing enhanced bioavailability. The emergence and application of biomaterials facilitated the introduction of cells and cellular membranes into nano delivery systems, enhanced biocompatibility, prolonged half-life, and facilitated the passage of physiological barriers, such as the blood-brain barrier (BBB) (Xia et al., 2020; Li et al., 2021; Xu et al., 2023). Additionally, in recent years, targeted delivery systems were increasingly preferred by researchers since their high efficiency and less side-effects, leading to better therapeutic performance. Specifically, the drug delivery could be more precise and efficient with the help of the selective banding between the biomaterials including antigens and antibodies, ligands and receptors, etc (Luo et al., 2023; Shang et al., 2023). Apart from safety and bioavailability, the functionality of the therapeutic agent is another determining factor for its clinical application. And in ICH, both the primary injury and the secondary injury are pivotal targets for clinical intervention. Specifically, increasingly preclinical drugs were applied to address the hematoma, ferroptosis, oxidative stress, inflammation, and apoptosis, etc (Xu et al., 2022; Zhang et al., 2023). And in their development, by realizing reduced nonspecific dispersion, controlled loading and release, and enhanced efficacy, these potential drug candidates demonstrated better performances against ICH. Further, benefiting from the outstanding engineerability of nanomaterials, comprehensive treatments for ICH was achieved through integrating pharmaceutical ingredients with different targets. Thus, it seemed to be more promising to develop multi-target drugs. Overall, after ICH, the heavy primary injury could be handled with medical and surgical managements (Puy et al., 2023), however, the secondary injury still needs to be prevented and intervened with effective approaches to improve the prognosis of ICH patients. Specifically, further clearing hematoma, inhibiting ferroptosis, oxidative damages, inflammatory responses, and apoptosis are pivotal targets. For these targets, besides therapeutic nanomaterials, more conventional preclinical drugs could be applied alone or combinedly after being improved in safety, bioavailability, and efficacy by nanotechnology such as modification and nano drug delivery systems (Yu et al., 2022). Apart from reducing injuries, the functional recovery after ICH also needs attention, thus, it is reasonable to improve present functional recovery therapies with nanotechnology (Gong et al., 2020).

Despite impressive achievements achieved with the support of nanotechnology in ICH, there are still significant challenges in translating research findings from the lab to clinical practice. Frankly, compared with cancer and other diseases, the exploration of mechanisms or therapies in ICH remains insufficient and needs more efforts (Figure 5C) (Shi et al., 2023). Thus, it is reasonable to learn from the latest advances in other diseases particularly cancer (Liang et al., 2020; Peng et al., 2022; Wang et al., 2022), hemorrhagic diseases (Zhuang et al., 2018), ischemic diseases (Chen et al., 2022; Zhang et al., 2022), and iron overloaded diseases (Yang et al., 2020; Zhao et al., 2021; Lu et al., 2022), etc. Besides, further exploration of nanotechnology to design multifunctional nanomaterials holds promising prospects. Polymeric nanomaterials with their diverse structures, offer great convenience in integrating with other therapeutic agents (Xu et al., 2018; Wang Y. et al., 2019; Zhang et al., 2021b). And for the sake of safety and convenience, many natural active ingredients including natural polyphenols (Zhu et al., 2023a), astaxanthin (Cai et al., 2022), vitamins (Lee et al., 2015), carotenoids (Harmatys et al., 2019), etc., are drawing increasingly attention in diseases therapies since they are both of natural therapeutic agents and outstanding building blocks for constructing multifunctional drugs (Figure 5C). For instance, the polyphenols, known for their versatile structures including catechol and pyrogallol moieties, can facilitate chemically crosslinking via oxidation and interact with nucleophilic groups through the oxidized quinone forms (Yang P. et al., 2021). In addition to covalently connection, polyphenols can also engage in hydrogen bonding, electrostatic interactions and *π*–*π* electron interactions (Zhang et al., 2021a). This versatility provides significant advantages in designing multi-target drugs by combining different agents. In addition to materials, further exploration of current nanotechnology such as post-synthetic modification, self-assembly and drug loading techniques, as well as employing biomaterials and developing innovative technologies, are critical in this field (Figure 5C). Lastly, besides therapy, prevention and diagnosis are of great importance in diseases management. As for ICH, its diagnosis as well as subsequent therapeutic strategy are largely depending on the imaging technology where nanotechnology is of great potential (Graeser et al., 2019; Szwargulski et al., 2020; Mazurek et al., 2021). Therefore, further developing nanomaterials based theragnostic agents is necessary for the generalization of precision medicine and personalized healthcare in ICH.
